# Retinal pigment epithelium melanin imaging using polarization-sensitive optical coherence tomography for patients with retinitis pigmentosa

**DOI:** 10.1038/s41598-022-11192-x

**Published:** 2022-05-03

**Authors:** Daiki Sakai, Seiji Takagi, Kota Totani, Midori Yamamoto, Mitsuhiro Matsuzaki, Masahiro Yamanari, Satoshi Sugiyama, Satoshi Yokota, Akiko Maeda, Yasuhiko Hirami, Michiko Mandai, Masayo Takahashi, Makoto Nakamura, Yasuo Kurimoto

**Affiliations:** 1 Department of Ophthalmology, Kobe City Eye Hospital, 2-1-8 Minatojima Minamimachi, Chuo-ku, Kobe-shi, Hyogo, 650-0047 Japan; 2 Department of Ophthalmology, Kobe City Medical Center General Hospital, Kobe, Japan; 3 Department of Surgery, Division of Ophthalmology, Kobe University Graduate School of Medicine, Kobe, Japan; 4 Department of Ophthalmology, Toho University Graduate School of Medicine, Tokyo, Japan; 5 Engineering Department, Tomey Corporation, Nagoya, Japan; 6 Vision Care Inc, Kobe, Japan

**Keywords:** Retinal diseases, Biomarkers

## Abstract

This study aimed to evaluate the distribution of retinal pigment epithelium (RPE) melanin in patients with retinitis pigmentosa (RP) using entropy measurements by custom-made polarization-sensitive optical coherence tomography (PS-OCT) images, and compare entropy with the intensity of short-wavelength (SW) and near-infrared (NIR) autofluorescence (AF). We retrospectively reviewed the retinal images, including PS-OCT, SW-AF, and NIR-AF of patients with RP who had a hyperautofluorescent ring on AF. A total of 12 eyes of 12 patients (8 women and 4 men; mean age: 37.9 years) were included. There was a strong positive correlation between entropy value and NIR-AF intensity (r = 0.626, p < 0.001), and there was a very weak negative correlation between entropy value and SW-AF (r = − 0.197, *p* = 0.001). The mean values of the entropy in the foveal, temporal (2 mm from the fovea), and nasal (2 mm from the fovea) sections were 0.41 (± 0.09), 0.29 (± 0.08), and 0.26 (± 0.08), respectively. The entropy was significantly higher in the foveal section than in the temporal and nasal sections (p = 0.002 and p = 0.003, respectively). There was no significant difference between the entropies values for the temporal and nasal sections (*p* = 0.157). Age, logMAR best-corrected visual acuity, ellipsoid zone width, and central retinal thickness were not correlated with foveal entropy. We presented RPE melanin imaging in patients with RP using PS-OCT for the first time. PS-OCT can be a useful tool for monitoring patients with RP.

## Introduction

Retinitis pigmentosa (RP) is the most common hereditary retinal dystrophy with a worldwide prevalence of approximately 1 in 4,000 individuals^1^. The clinical course of RP is characterized by night blindness and progressive loss of the visual field due to degeneration and loss of photoreceptors. For typical RP, rod photoreceptors are primarily affected, followed by the loss of secondary cone photoreceptors and retinal pigment epithelium (RPE). While, RPE dysfunction leads to photoreceptor function impairment and results in the loss of photoreceptors in RP with several causative genes, including MER proto-oncogene, tyrosine kinase (*MERTK*), *RPE65*, *LRAT*, and bestrophin 1 (*BEST1*)^2^.

The RPE is an indispensable partner of the neural retina. Located between the neural retina and choroid, the RPE plays an essential role in the survival and function of photoreceptors^3^. Three major pigments, lipofuscin, melanin, and melanolipofuscin, are present in the RPE. Clinically, autofluorescence (AF) imaging has been used to evaluate RPE. Short-wavelength AF (SW-AF; 488 nm excitation) originates from lipofuscin in the RPE^4^, whereas near-infrared AF (NIR-AF; 785 nm excitation) originates from melanin in the RPE, with a smaller contribution from the choroid^5^. Characteristic hyperautofluorescent rings are often observed in patients with RP, and this ring shows progressive constriction with disease progression^6^. The hyperautofluorescent ring is proposed as a useful indicator for monitoring RP, because previous studies have shown that the ring is correlated with retinal function evaluated by various examinations, including perimetry^7,8^ and electroretinogram^9^. While SW-AF is mainstream for monitoring RP, especially in clinical settings, recent studies have proposed the usefulness of NIR-AF. NIR-AF findings are well-correlated with SW-AF findings, and they could be used to detect a unveil process in the progression of RP, which precedes that detected by SW-AF^8,10^. Hence, the evaluation of melanin in the RPE is worth noting for further understanding of RP progression.

Polarization-sensitive optical coherence tomography (PS-OCT) is a functional extension of OCT that measures the polarization properties of ocular tissues^11^. One of the characteristic polarization properties is depolarization by melanin. Although its physical mechanism is not fully understood yet, it has been suggested that multiple light scattering and scattering from unevenly nonspherical particles might cause the depolarization. PS-OCT can visualize melanin-containing structures such as the RPE, choroid, and pigment epithelium of the iris. In particular, PS-OCT is promising for the imaging of melanin in the RPE. To quantify melanin in the RPE, the degree of polarization uniformity (DOPU) has been determined and successfully utilized in previous studies^12–14^. More recently, polarimetric entropy has been suggested as one of improved approaches to quantify the depolarization, which has several technical advantages including insensitivity to the incident state of polarization and availability of noise-bias correction^15,16^. In this study, we aimed to evaluate the distribution of RPE melanin in patients with typical RP with a hyperautofluorescent ring by using entropy measurements by PS-OCT and compare the entropy with the intensities of SW- and NIR-AF.

## Methods

### Study design

This retrospective study adhered to the principles of the Declaration of Helsinki and was approved by the Medical Ethics Committee of the Kobe City Medical Center General Hospital (Kobe, Japan). All participating patients provided oral informed consent to participate in this study. The ethics committee waived written informed consent for this observational study involving the use of PS-OCT images and other medical records, which was harmless to the patients.

### Patients

We reviewed the medical records of patients diagnosed with RP who were followed up at Kobe City Eye Hospital between December 2019 and March 2020. RP was diagnosed according to the guidelines of the Japanese Ophthalmological Society^17^ based on clinical history, the appearance of the fundus, visual fields, and full-field electroretinogram results. The inclusion criteria were as follows: (1) patients who underwent multimodal retinal imaging, including PS-OCT, SW-AF, and NIR-AF, and (2) patients with a hyperautofluorescent ring on AF. The presence of a hyperautofluorescent ring was determined by agreement between the two observers (D.S. and M. Matsuzaki). The exclusion criteria included retinal confounding diseases such as epiretinal membrane and cystoid macular edema, media opacification that impaired image quality, and poor image quality due to unstable fixation. Finally, 12 patients from 12 unrelated families were included in the study. If both eyes of one patient were available, only the right eye of a patient was selected and included in the analysis.

### Multimodal retinal imaging

#### PS-OCT images

We used a custom-made PS-OCT device (Tomey, Aichi, Japan) in this study. The details of the system has been described previously^16^. The system measured depth-resolved Jones matrices, which described the polarization property of the sample and were the raw data of our PS-OCT for extracting OCT intensity and polarization-sensitive contrasts. The raster-scanned images (1024 A-scans × 256 B-scans) were obtained, covering a macular area of 6 × 6 mm (approximately 20 × 20 degrees). The size of each pixel in the B-scan was H: 6/1024A = 5. 86 µm and V: 4.2 µm (refractive power for calculating the depth resolution in the tissue was 1.38^18^).

### AF images

Both SW-AF and NIR-AF images were obtained using the Heidelberg Spectralis HRA (Heidelberg Engineering, Heidelberg, Germany). The SW-AF images were acquired using an excitation filter of 486 nm and a barrier filter of 500 nm. The NIR-AF images were acquired using an excitation filter of 786 nm and a barrier filter of 830 nm. The field of view was set to 30 × 30 degrees, and the resolution was set to 768 × 768 pixels.

### Image analysis

#### RPE-melanin and retinal evaluation with PS-OCT images

A B-scan image along the horizontal meridian through the fovea obtained using PS-OCT was selected for each eye. The fovea was identified by referring to the foveal bulge. If the foveal bulge was absent, the fovea was identified by comprehensively considering the fixation point or the point of maximum ONL thickness and minimum inner retinal layer thickness. Manual segmentation was performed to identify the RPE line referring to the intensity OCT by two retina image specialists (K.T. and D.S.). The spatial randomness of the retinal polarization property was parameterized as polarimetric entropy with a range of “0” (completely uniform) to “1” (completely random polarization)^15^. The polarimetric entropy was calculated for each pixel, and the kernel size for calculation was set to 5 × 11 pixels. It has been previously shown that the polarimetric entropy is correlated with the concentration of melanin granules^16^. Entropy at the RPE line was defined as the average within six pixels (25 μm) of the segmentation line. Assuming that each B-scan image width was equivalent to 6 mm, we separated the RPE line from the foveal section with 0.25 mm width and sections extending to the temporal and nasal sections with the foveal section at 0.25 mm intervals. The average value of entropy at the RPE line in each section was plotted on a line chart to present the distribution of RPE melanin for each eye. In the line chart, the values in the section located on the temporal side were always displayed on the left side of the chart, and the values in the section located on the nasal side were always displayed on the right side of the chart; the line charts of the left eyes were laterally reversed for consistency with those of the right eyes. The entropy values for the temporal and nasal sections (defined as the section located 2 mm from the fovea, respectively), as well as those for the foveal section, were collected and used to investigate the distribution of RPE-entropy in patients with RP. Additionally, the ellipsoid zone (EZ) width and central retinal thickness (CRT) were manually measured on the intensity OCT images. The length values that were corrected based on the total imaging range of 6 mm were used. The EZ width was defined as the distance between the temporal and nasal borders on the horizontal image of the EZ, where the EZ line disappeared. CRT was defined as the length from the vitreoretinal interface to the inner border of the RPE. The presence of EZ disruption (disruption of the remnant EZ line on the horizontal intensity OCT images) was also recorded.

### AF image processing

First, the maximum intensity projection was performed for raster-scanned PS-OCT images using the original software. The SW- and NIR-AF images were registered by referring to several characteristic points, such as vessel bifurcations. We used the bUnwarpJ package in the ImageJ program for image registration^19^. The AF intensity was measured along the horizontal line corresponding to the RPE line in the PS-OCT images. Finally, the horizontal line on the AF images was separated into sections with widths of 0.25 mm, and the average value for each section was displayed on the line chart in the same manner as the PS-OCT analysis.

### Statistical analysis

Spearman’s rank correlation coefficient was used to determine correlations between the entropy and SW- and NIR-AF intensities. The values for all the sections from all participants were assessed together for the correlation analysis. Since the AF intensities of the participants were difficult to compare, a quantitative AF approach that uses an internal AF reference has been proposed^20^. In this study, we used a simplified internal AF reference for the correlation analysis. In short, the maximum intensity was regarded as “1,” and the minimum intensity was regarded as “0” for each eye. The entropy values of the three sections were compared using the Wilcoxon rank-sum test with Bonferroni correction. Correlations between the entropy values in the foveal section and clinical findings were assessed using Spearman’s rank correlation coefficient. The best-corrected visual acuity (BCVA) of the patients was obtained using Landolt C charts and converted to the logarithm of the minimum angle of resolution (logMAR) equivalent for statistical comparisons. All statistical analyses were conducted using the SPSS statistical software package (version 28; SPSS Inc., Chicago, IL, USA).

### Ethics approval

All procedures performed in studies involving human participants were in accordance with the ethical standards of the Kobe City Medical Center General Hospital (Kobe, Japan) and the principles of the 1964 Declaration of Helsinki and its later amendments or comparable ethical standards.

### Consent to participate/ Consent for publication

All participating patients provided oral informed consent to participate in this study. The Medical Ethics Committee of the Kobe City Medical Center General Hospital (Kobe, Japan) waived written informed consent for this observational study involving the use of PS-OCT images and other medical records, which was harmless to the patients. The concept of PS-OCT and the purpose of the study was adequately explained, and oral informed consent was recorded on the medical records before PS-OCT examination.

## Results

**Figure 1:**
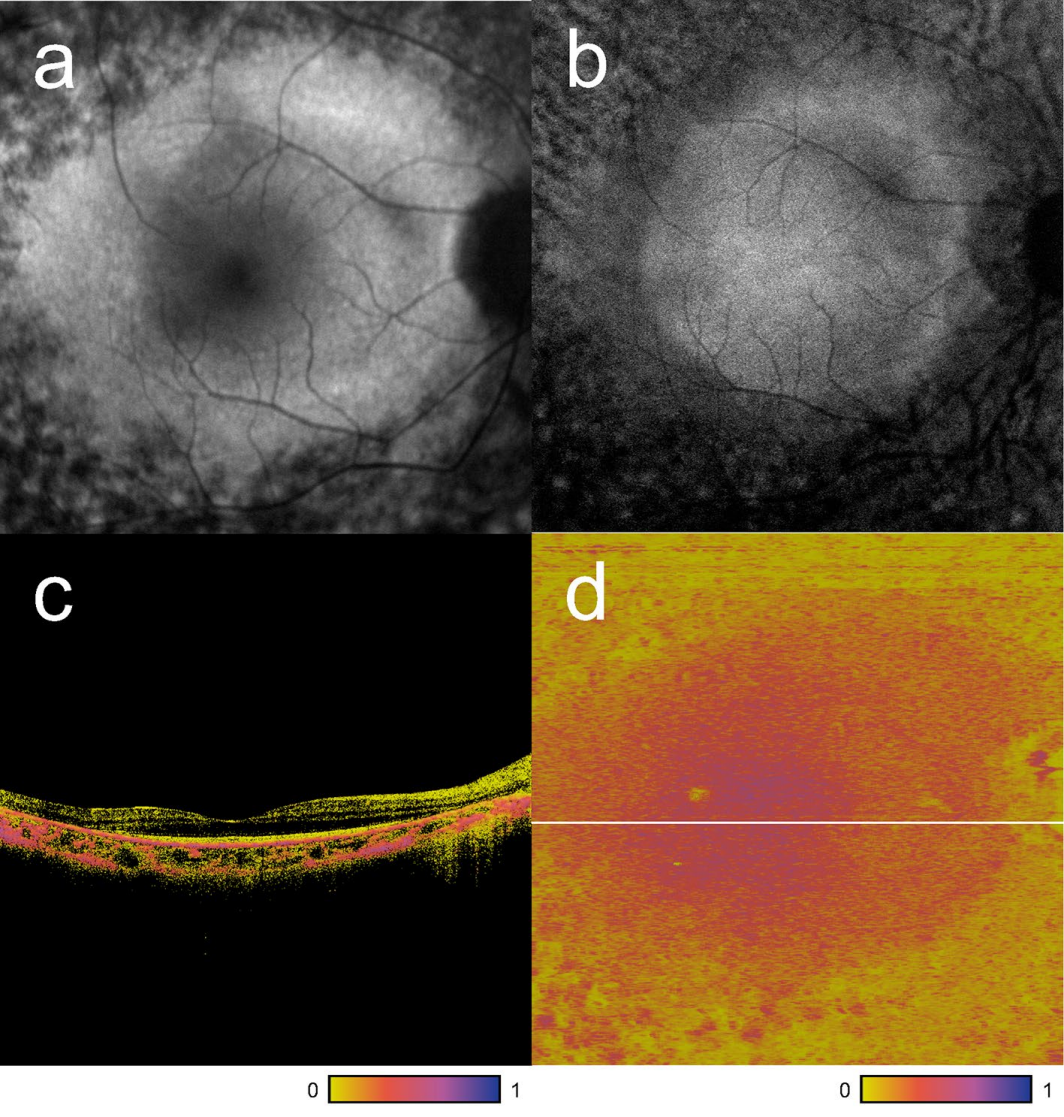
Representative images of multimodal retinal imaging. (**a**) Short-wavelength autofluorescence (AF). (**b**) Near-infrared AF image. (**c**) Polarization-sensitive optical coherence tomography (PS-OCT) B-scan image along the horizontal meridian through the fovea. (**d**) En face PS-OCT image which is extracted at the RPE level of patient 1. The white line is a trace line of the B-scan image (**c)**.

**Figure 2:**
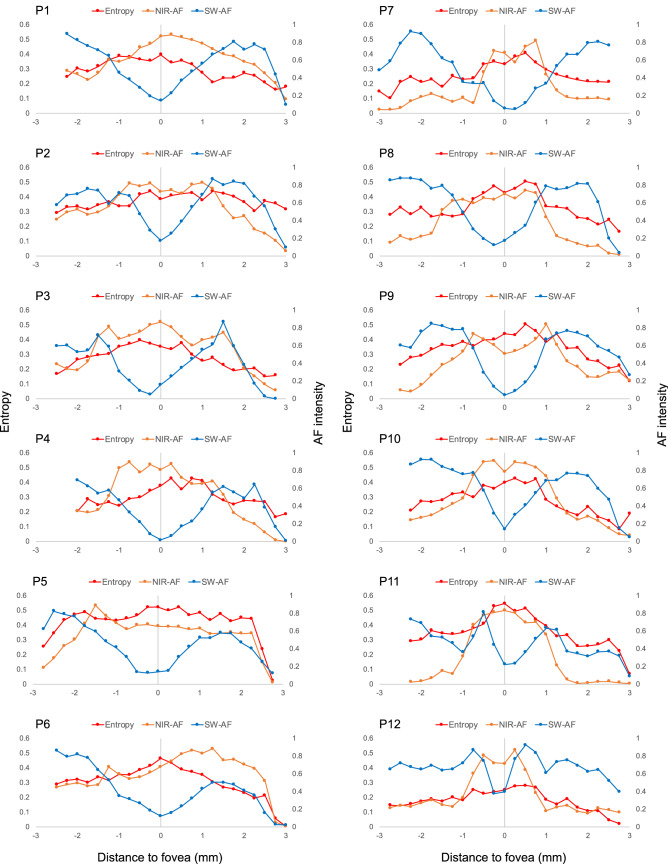
Distribution of entropy values, near-infrared and short-wavelength autofluorescence intensities for each patient.

**Figure 3:**
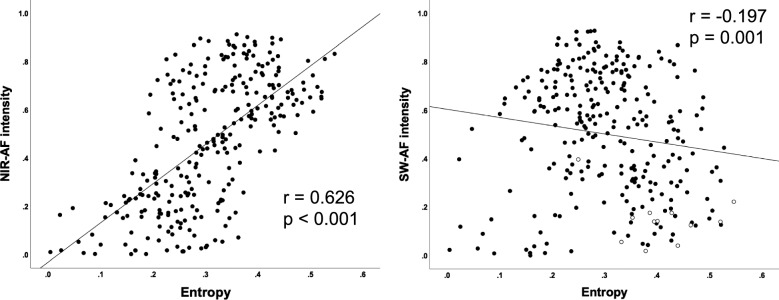
Scatter plots showing the relationship between entropy value and autofluorescent (AF) intensities. (**a**) Relationship between the entropy values and near-infrared AF intensities. (**b**) Relationship between the entropy values and short-wavelength AF intensities. The plot on the foveal section of each patient was colored in white.

**Figure 4:**
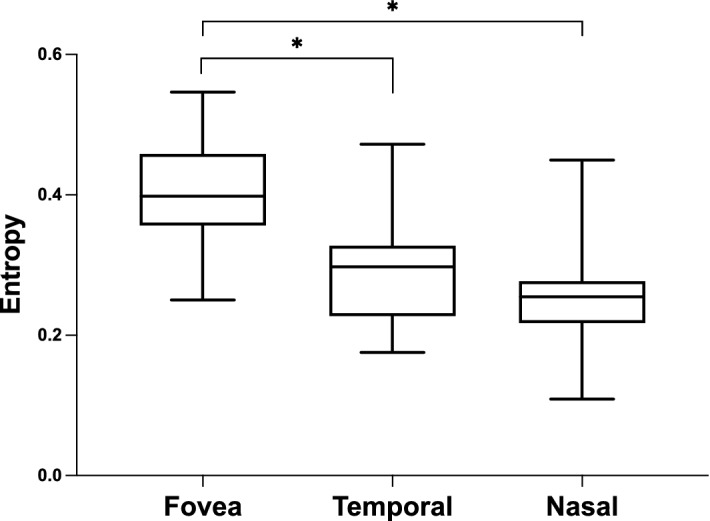
Box and whisker plots of entropy for three sections. The horizontal line in the box represents the median. The top and bottom sections of the box represent the upper and lower quartiles, respectively. The whisker above the box represents the values within 1.5 times the interquartile range plus the upper quartile, and the whisker below the box represents the values within 1.5 times the interquartile range minus the lower quartile. *Statistically significant at *P* < 0.05.

**Figure 5:**
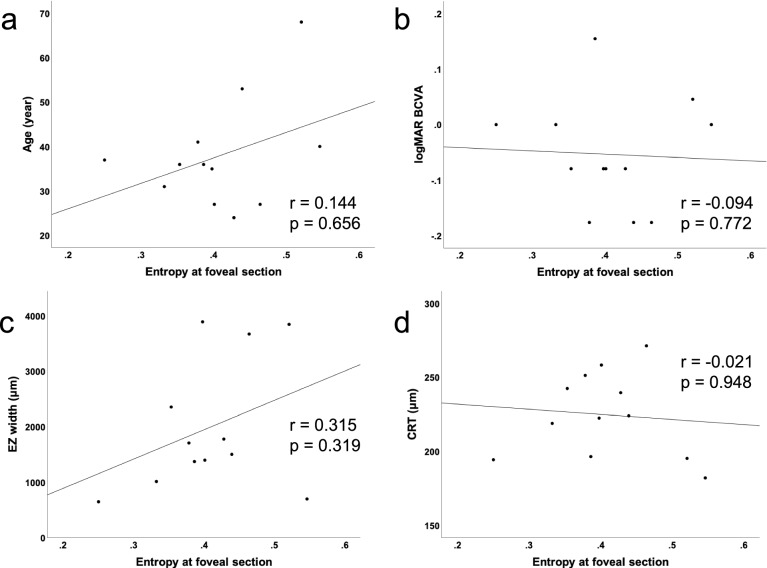
Scatter plots showing the relationship between entropy values at the fovea and other clinical findings. (**a**) Relationship between the entropy at the fovea and age. (**b**) Relationship between the entropy at the fovea and logMAR best-corrected visual acuity. (**c**) Relationship between the entropy at the fovea and the ellipsoid zone width. (**d**) Relationship between the entropy at the fovea and central retinal thickness.

The patients’ demographic data and clinical findings are summarized in Table 1. The mean (± standard deviation) age was 37.9 (± 12.2) years. Eight of the 12 patients were women. All the patients were Japanese. Causative genes were identified in seven patients. Among them, four patients had *EYS* mutations, and three patients had *USH2A* mutations. The mean logMAR BCVA was –0.05 (± 0.10). The mean EZ width was 1991 ± 1187 μm, and the mean CRT was 225 (± 28.6) μm. None of the patients had an EZ disruption at the fovea. Figure 1 shows representative images of multimodal retinal imaging obtained from patient 1. Figure 2 shows the distribution of the entropy values, NIR-AF intensities, and SW-AF intensities for each patient. The details of measurement results can be found as Supplementary Table S1 online. There was a significant correlation between the entropy value and NIR-AF intensity (r = 0.626, p < 0.001). There was also a significant correlation between the entropy value and SW-AF (r = -0.197, p = 0.001) (Fig. 3). The mean entropy values for the foveal, temporal (2 mm from the fovea), and nasal (2 mm from the fovea) sections were 0.41 (± 0.09), 0.29 (± 0.08), and 0.26 (± 0.08), respectively. The distribution of entropy in each section is shown in Fig. 4. The entropy was significantly higher in the foveal section than in the temporal and nasal sections (*p* = 0.002 and *p* = 0.003, respectively). There was no significant difference between the entropy values for the temporal and nasal sections (p = 0.157). The relationship between entropy in the foveal section and the clinical findings of the patients is shown in Fig. 5. Age, logMAR BCVA, EZ width, and CRT were not correlated with foveal entropy.

**Table 1:** Demographic and clinical findings of patients.

Patient	Age	Sex	Eye	Causative genes	Lens status	BCVA (decimal)	Entropy in the foveal section	Entropy in the temporal section	Entropy in the nasal section	EZ width (μm)	CRT (um)
1	35	F	OD	*EYS*	phakia	1.2	0.40	0.30	0.27	3890	223
2	36	F	OD	*USH2A*	phakia	0.7	0.39	0.34	0.37	1375	197
3	36	F	OD	*EYS*	phakia	1.2	0.35	0.26	0.20	2355	242
4	41	F	OD	NA	phakia	1.5	0.38	0.21	0.28	1710	251
5	68	F	OD	*USH2A*	pseudophakia	0.9	0.52	0.47	0.45	3845	195
6	27	M	OD	*EYS*	phakia	1.5	0.46	0.32	0.23	3670	271
7	31	M	OS	NA	phakia	1	0.33	0.22	0.21	1015	219
8	24	F	OD	*USH2A*	phakia	1.2	0.43	0.33	0.25	1780	240
9	53	F	OD	NA	phakia	1.5	0.44	0.29	0.27	1505	224
10	27	M	OD	*EYS*	phakia	1.2	0.40	0.27	0.24	1400	258
11	40	F	OD	NA	phakia	1	0.55	0.30	0.26	700	182
12	37	M	OD	NA	phakia	1	0.25	0.18	0.11	650	194

NA not available, BCVA best-corrected visual acuity, EZ ellipsoid zone, CRT central retinal thickness.

## Discussion

In this study, we introduced RPE-melanin imaging for patients with RP using PS-OCT for the first time. We compared the PS-OCT findings “entropy” with the NIR and SW-AF findings. There was a strong positive correlation between entropy and NIR-AF intensity. While, there was a very weak negative correlation between entropy and the SW-AF intensity. The NIR- and SW-AF imaging, which represent melanin and lipofuscin distribution in the fundus, have been used to monitor the disease progression of RP. In particular, the use of NIR-AF has been expanding following its application to multiple clinical studies^8,10^. PS-OCT may have potential as an additional tool for monitoring patients with RP.

PS-OCT showed RPE-melanin alterations that were consistent with the NIR-AF findings. We observed the distribution of melanin specific to the RPE using PS-OCT cross-sectional images. Although RPE containing pigments are the major source of AF, a lack of topographic information is known as one of the weak points of AF imaging. In addition to the RPE layer, the photoreceptor layer and choroid may also contribute to AF. Thus, we believe that PS-OCT can detect RPE-melanin changes more precisely. In most patients in our case series, melanin distributions provided by PS-OCT and NIR-AF showed some degree of similarity. However, we found several discrepancies between the findings of the two modalities. First, in some patients (P 7, 8, 11), NIR-AF intensity significantly decreased in the peripheral part of the images as compared to entropy. In prior research, Duncker et al. compared NIR- and SW-AF in patients with RP and reported that the abrupt loss of signal outside the hyperautofluorescent ring was characteristic of NIR-AF^8^. However, it seems that the decrease in entropy toward the periphery was relatively moderate in our results. There may be a difference in the detection limit of melanin by PS-OCT and NIR-AF, since these two modalities use different methods to observe melanin. Continuous RPE changes are considered to occur in patients with RP, which corresponds to the slow progression of retinal degeneration^21^. Thus, if PS-OCT has good detectability of RPE melanin, it could be a useful tool for the detailed evaluation of RPE conditions. Second, in some patients (P 3, 6, 9), the distributions of NIR-AF intensity have two sharp peaks corresponding to the well-known hyperautofluorescent ring. However, we did not find apparent peaks of entropy in the same part of the same patient. The hyperautofluorescent ring is an important finding in patients with RP, which is observed in the transition zone between healthy and degenerate retinas, but its origin has not been fully understood. Considering that the PS-OCT findings in this study are specific to the RPE layer, our results suggest that the hyperautofluorescent ring may have multiple contributors, including not only the RPE layer but also the photoreceptor layer and choroid. Histologically, the death of photoreceptors and RPE cells has been observed as a common pathology in the disease progression of RP. Photoreceptors and RPE cells are indispensable to each other, and whether the photoreceptors or RPE cells impairment comes first during the development of RP has not been established. Recent advancements in genetic analyses have revealed that mutations in some genes that are expressed in RPE, such as *MERTK*, *RPE65*, *LRAT*, or *BEST1*, cause retinal degeneration. Therefore, RPE cells have become an important target for the development of treatments for RP. RPE replacement therapies are among the most attractive strategies and have already been applied to patients with RPE degeneration, such as age-related macular degeneration (AMD)^22^ and Stargardt disease^23^. For optimal indication and reliable outcome measurements for RPE replacement therapy, a precise evaluation of RPE conditions is crucial. Our group have previously reported that PS-OCT may be effective to monitor the survival and melanin content of transplanted human induced pluripotent stem cell-derived RPE in patient with AMD^24,25^. Photoreceptor cell transplantation is another promising strategy. The importance of RPE evaluation is also applied to photoreceptor cell transplantation because the healthiness of the underlying RPE cells is theoretically essential for successful treatment. Moreover, both photoreceptors and RPE cells are the main targets in gene therapy for retinal degeneration^26^. In short, layer-specific evaluation of the anatomical and functional conditions of the retina has become more important in the new era of retinal cell therapy. NIR-AF imaging has been established as the method for the evaluation of the RPE. Although the NIR-AF mainly originates from melanin in the RPE, there is a varying degree of choroidal or other retinal layer contributions, which makes it difficult to interpret the findings. We consider that PS-OCT has a potential to evaluate RPE conditions simply by utilizing the ability of retinal layer stratification. This study observed the RPE layer in patients with RP and found a moderate decrease in RPE-melanin toward the periphery without apparent peaks. We analyzed these PS-OCT findings by comparing them with those of NIR-AF. In the future, the assessment of choroidal melanin using PS-OCT may be useful for explaining the differences between the findings of the two modalities.

We also compared the entropy with the SW-AF intensity to investigate the relationship between the two major pigments in the RPE; melanin and lipofuscin. As shown in the results, there was a very weak negative correlation between entropy and SW-AF intensity. The SW-AF intensity in the foveal section is generally low (hypofluorescence) because of the high density of macular pigment that blocks the normal autofluorescence of the RPE (Fig. 3b). On the contrary, entropy is high in the foveal section because of the larger number of melanin granules in each RPE cell or the denser packing of RPE cells at the macula^27^. This discrepancy between the two values of entropy in the fovea may be attributed to the negative correlation. Thus, our results of the very weak negative correlation between entropy and SW-AF intensity may have been affected by the methodological problem of SW-AF, besides a possible correlation between melanin and lipofuscin. So far, the molecular correlations between RPE pigments are not fully understood. The RPE contains the third pigment of melanolipofuscin, which is composed of a melanin core and a lipofuscin shell. Note that PS-OCT may detect pure melanin granules, as well as melanolipofuscin. Further investigations of the findings of PS-OCT and other modalities are required to further understand how the distribution of RPE pigments changes with the progression of RP.

We determined the distributions of entropy in three (fovea, temporal, and nasal) sections, as the quantitative evaluation of the melanin in the RPE in patients with RP. We found that the entropy was significantly higher in the foveal section than in other sections, whereas there was no significant difference between the values of entropy in the temporal and nasal sections. The diameters of the hyperfluorescent rings varied among patients, and this may be a limitation; however, we expect that our results will provide fundamental insights into the effects of RP on RPE entropy. In this study, we included only patients with characteristic hyperautofluorescent rings visible on AF. Since the hyperautofluorescent ring is recognized as the boundary between the healthy and unhealthy retina, the area within the ring theoretically has a preserved function. Our patients had preserved visual acuity, and no patients had EZ disruption at the fovea. Among these patients, entropy at the foveal section varied between 0.25 and 0.52. Fujita et al. reported an almost similar range of entropy around the fovea in healthy participants^27^. They reported a positive correlation between age and entropy around the fovea. We also investigated the relationship between foveal entropy and other clinical findings, such as age, visual acuity, and OCT parameters. However, none was correlated with foveal entropy. Shu et al. suggested the existence of individual differences in the distribution of RPE-melanin using photoacoustic microscopy^28^. Given that the entropy measurement by PS-OCT has could be a quantitative evaluation tool for RPE conditions, it is essential to understand individual differences and associating factors of entropy. Further investigations of entropy measurements involving healthy and diseased eyes are warranted.

This study has limitations. This case series was retrospective and included a limited number of eyes. We included only patients with a hyperautofluorescent ring. Murakami et al. reported that the hyperautofluorescent ring was observed in 59% of patients with RP^29^. Our findings from this study may not be applicable to all RP cases. Moreover, our study lacked functional evaluation of the retina, including visual field testing or electroretinogram. For the utilization of PS-OCT and entropy measurements in clinical practice, their relationships with retinal function need to be investigated. Additionaly, regarding the technical aspect, our PS-OCT is tuned to scan a macular area of 6 × 6 mm for the emmetropic eye with an axial length of 24.0 mm. We did not have any available correction for axial length variations in our prototype device, which should be considered in the future. Finally, genetic information was insufficient in our series. The variants of patients are considered important for interpreting RPE-melanin findings. Especially, patients with mutations that affect RPE should be included in future studies.

In conclusion, we presented an initial case series of RPE-melanin imaging using PS-OCT in patients with RP. Entropy measurements obtained from the PS-OCT images were highly correlated with NIR-AF intensity. The potentials for layer-specific and quantitative evaluation are considered as advantages of PS-OCT as compared to AF imaging. PS-OCT may be a useful tool for monitoring patients with RP.

## Supplementary Information

Supplementary Information.

## Data Availability

All data generated or analysed during this study are included in this published article and its supplementary information file.
