# Supplementary material for: Retinal pigment epithelium melanin imaging using polarization-sensitive optical coherence tomography for patients with retinitis pigmentosa
Source: Sci Rep. 2022 May 3;12:7115. doi: 10.1038/s41598-022-11192-x (PMC9065024; doi:10.1038/s41598-022-11192-x)
Supplement: Supplementary file 1 — Supplementary Information. [file 41598_2022_11192_MOESM1_ESM.docx]

**Retinal pigment epithelium melanin imaging using polarization-sensitive optical coherence tomography for patients with retinitis pigmentosa**

Daiki Sakai*^1, 2, 3^; Seiji Takagi^4^; Kota Totani^5^; Midori Yamamoto^1^; Mitsuhiro Matsuzaki^1, 2^; Masahiro Yamanari^5^; Satoshi Sugiyama^5^; Satoshi Yokota^1, 2^; Akiko Maeda^1^; Yasuhiko Hirami^1, 2^; Michiko Mandai^1^; Masayo Takahashi^1, 6^; Makoto Nakamura^3^; Yasuo Kurimoto^1, 2^

**Supplementary Table S1 Entropy and near-infrared/short-wave autofluorescence intensities measured for patients in the study.**

|  | Distance to fovea (mm) | Entropy | NIR-AF intensity | SW-AF intensity |
| --- | --- | --- | --- | --- |
| P1 | -2.25 | 0.246347471 | 0.47926645 | 0.89165733 |
|  | -2 | 0.303309133 | 0.43949372 | 0.825028 |
|  | -1.75 | 0.282681176 | 0.37609659 | 0.75858527 |
|  | -1.5 | 0.317091536 | 0.45325 | 0.71192609 |
|  | -1.25 | 0.361601847 | 0.60386782 | 0.64427024 |
|  | -1 | 0.388431755 | 0.58173834 | 0.45604703 |
|  | -0.75 | 0.38218363 | 0.62041447 | 0.37933931 |
|  | -0.5 | 0.365230507 | 0.74172638 | 0.28611422 |
|  | -0.25 | 0.355671925 | 0.78319378 | 0.19148936 |
|  | 0 | 0.394835791 | 0.86862051 | 0.1399776 |
|  | 0.25 | 0.342467321 | 0.88436992 | 0.22424412 |
|  | 0.5 | 0.356382208 | 0.85805413 | 0.36982083 |
|  | 0.75 | 0.330695348 | 0.82685377 | 0.47172452 |
|  | 1 | 0.272084946 | 0.78747996 | 0.56177676 |
|  | 1.25 | 0.208532864 | 0.72328529 | 0.66340053 |
|  | 1.5 | 0.237609493 | 0.66766328 | 0.72293765 |
|  | 1.75 | 0.239972652 | 0.64423864 | 0.80711086 |
|  | 2 | 0.273280117 | 0.58183822 | 0.71603211 |
|  | 2.25 | 0.254009693 | 0.54814593 | 0.77519597 |
|  | 2.5 | 0.206606163 | 0.44926256 | 0.71659202 |
|  | 2.75 | 0.160282209 | 0.33702153 | 0.43850316 |
|  | 3 | 0.177969734 | 0.15610048 | 0.09518477 |
| P2 | -2.5 | 0.292010834 | 0.41296502 | 0.57767478 |
|  | -2.25 | 0.332720537 | 0.49608066 | 0.68775566 |
|  | -2 | 0.336364652 | 0.52693789 | 0.7027543 |
|  | -1.75 | 0.315510574 | 0.46870724 | 0.75756749 |
|  | -1.5 | 0.347059762 | 0.49720045 | 0.73820562 |
|  | -1.25 | 0.368176323 | 0.56028373 | 0.5924916 |
|  | -1 | 0.337901804 | 0.69540873 | 0.7118444 |
|  | -0.75 | 0.338190546 | 0.81933557 | 0.6812108 |
|  | -0.5 | 0.418673845 | 0.79134005 | 0.47250251 |
|  | -0.25 | 0.43833942 | 0.82232176 | 0.28661031 |
|  | 0 | 0.386448747 | 0.72763471 | 0.1758931 |
|  | 0.25 | 0.409426239 | 0.74405875 | 0.2527952 |
|  | 0.5 | 0.421987211 | 0.70287421 | 0.38941914 |
|  | 0.75 | 0.42855005 | 0.80739082 | 0.55431325 |
|  | 1 | 0.378918108 | 0.82866741 | 0.69402784 |
|  | 1.25 | 0.439329593 | 0.75986066 | 0.8682847 |
|  | 1.5 | 0.424717502 | 0.55941273 | 0.802018 |
|  | 1.75 | 0.403898942 | 0.4271495 | 0.84155986 |
|  | 2 | 0.365135742 | 0.45253206 | 0.81619853 |
|  | 2.25 | 0.306285991 | 0.29973875 | 0.67521134 |
|  | 2.5 | 0.372499979 | 0.25693666 | 0.55967641 |
|  | 2.75 | 0.357464029 | 0.17357223 | 0.30242705 |
|  | 3 | 0.318406044 | 0.05897723 | 0.09435506 |
| P3 | -2.5 | 0.166186744 | 0.39043826 | 0.59398088 |
|  | -2.25 | 0.205982354 | 0.33370504 | 0.60069604 |
|  | -2 | 0.26429639 | 0.32085586 | 0.52613557 |
|  | -1.75 | 0.282298711 | 0.42195765 | 0.54343733 |
|  | -1.5 | 0.295059813 | 0.70734395 | 0.71442973 |
|  | -1.25 | 0.30483502 | 0.81236578 | 0.58614711 |
|  | -1 | 0.352569255 | 0.67564431 | 0.30853066 |
|  | -0.75 | 0.372324981 | 0.71060137 | 0.20054545 |
|  | -0.5 | 0.395613481 | 0.75720047 | 0.0895654 |
|  | -0.25 | 0.372982308 | 0.83399156 | 0.04809428 |
|  | 0 | 0.35343854 | 0.86484644 | 0.15635199 |
|  | 0.25 | 0.334113476 | 0.80901769 | 0.24963691 |
|  | 0.5 | 0.377669669 | 0.69383133 | 0.34891176 |
|  | 0.75 | 0.2983731 | 0.59921588 | 0.44818662 |
|  | 1 | 0.256420902 | 0.66354949 | 0.55118071 |
|  | 1.25 | 0.277538324 | 0.69253433 | 0.61291685 |
|  | 1.5 | 0.22762389 | 0.74525658 | 0.86908816 |
|  | 1.75 | 0.191924349 | 0.58509983 | 0.59361881 |
|  | 2 | 0.203614766 | 0.33687235 | 0.37885682 |
|  | 2.25 | 0.205832661 | 0.25380773 | 0.16633423 |
|  | 2.5 | 0.150816947 | 0.15997596 | 0.0237749 |
|  | 2.75 | 0.158554449 | 0.09084579 | 0 |
| P4 | -2 | 0.205678325 | 0.3446225 | 0.69430896 |
|  | -1.75 | 0.287631763 | 0.32711678 | 0.62558837 |
|  | -1.5 | 0.247739368 | 0.35697948 | 0.54505777 |
|  | -1.25 | 0.267318929 | 0.51395888 | 0.57869063 |
|  | -1 | 0.242425499 | 0.82848989 | 0.46709456 |
|  | -0.75 | 0.290131411 | 0.89473705 | 0.32991014 |
|  | -0.5 | 0.299324489 | 0.78032052 | 0.21874198 |
|  | -0.25 | 0.345021602 | 0.87002308 | 0.08164313 |
|  | 0 | 0.378604139 | 0.8123571 | 0.01848524 |
|  | 0.25 | 0.427640273 | 0.87448529 | 0.06161746 |
|  | 0.5 | 0.352176284 | 0.72322674 | 0.17150193 |
|  | 0.75 | 0.427197675 | 0.6496569 | 0.22901155 |
|  | 1 | 0.413508761 | 0.65343261 | 0.36713736 |
|  | 1.25 | 0.318663152 | 0.68238001 | 0.55353014 |
|  | 1.5 | 0.2797601 | 0.53192229 | 0.62088146 |
|  | 1.75 | 0.253525364 | 0.32643028 | 0.55806591 |
|  | 2 | 0.278128924 | 0.24748289 | 0.48857512 |
|  | 2.25 | 0.275033045 | 0.19942796 | 0.64261874 |
|  | 2.5 | 0.269583382 | 0.1012586 | 0.38818999 |
|  | 2.75 | 0.166495047 | 0.01647598 | 0.16277279 |
|  | 3 | 0.18660983 | 0.00171625 | 0.01001284 |
|  | 3.25 | 0.08959062 | 0.0020595 | 0.0084724 |
|  | 3.5 | 0.158120717 | 0.001373 | 0.01026958 |
| P5 | -2.75 | 0.253514933 | 0.18560895 | 0.61814586 |
|  | -2.5 | 0.344399619 | 0.29061785 | 0.82345865 |
|  | -2.25 | 0.432533051 | 0.4310535 | 0.78947368 |
|  | -2 | 0.472041058 | 0.50343252 | 0.76360902 |
|  | -1.75 | 0.488004877 | 0.68336301 | 0.65323308 |
|  | -1.5 | 0.443844395 | 0.89083822 | 0.59598977 |
|  | -1.25 | 0.439979285 | 0.77464195 | 0.48431068 |
|  | -1 | 0.430261892 | 0.68971948 | 0.41593985 |
|  | -0.75 | 0.444716131 | 0.62123908 | 0.30646617 |
|  | -0.5 | 0.466406888 | 0.66624287 | 0.13593985 |
|  | -0.25 | 0.521909186 | 0.67387066 | 0.12661654 |
|  | 0 | 0.520981228 | 0.65395372 | 0.13924812 |
|  | 0.25 | 0.499743275 | 0.65132639 | 0.1524812 |
|  | 0.5 | 0.521211008 | 0.64683445 | 0.30827068 |
|  | 0.75 | 0.467979195 | 0.62064584 | 0.42115278 |
|  | 1 | 0.484196315 | 0.6287821 | 0.52240602 |
|  | 1.25 | 0.432273348 | 0.56826852 | 0.52009985 |
|  | 1.5 | 0.47568493 | 0.57784554 | 0.57884722 |
|  | 1.75 | 0.426231532 | 0.58581238 | 0.57323308 |
|  | 2 | 0.449557247 | 0.57233664 | 0.47057654 |
|  | 2.25 | 0.442852089 | 0.57547251 | 0.40130346 |
|  | 2.5 | 0.236365816 | 0.24637681 | 0.25453654 |
|  | 2.75 | 0.025013656 | 0.01703534 | 0.11879699 |
| P6 | -2.5 | 0.288837435 | 0.44944179 | 0.86219171 |
|  | -2.25 | 0.315103043 | 0.47878788 | 0.79328757 |
|  | -2 | 0.323540214 | 0.49601276 | 0.82201882 |
|  | -1.75 | 0.303097043 | 0.46347687 | 0.78032037 |
|  | -1.5 | 0.33874146 | 0.47719298 | 0.64302059 |
|  | -1.25 | 0.317344708 | 0.67623601 | 0.52996018 |
|  | -1 | 0.357800242 | 0.59702281 | 0.35341978 |
|  | -0.75 | 0.354309064 | 0.54417863 | 0.31578947 |
|  | -0.5 | 0.387810186 | 0.56438067 | 0.26646326 |
|  | -0.25 | 0.415552514 | 0.61573627 | 0.18459191 |
|  | 0 | 0.464214752 | 0.68176501 | 0.12509535 |
|  | 0.25 | 0.43469698 | 0.74364699 | 0.16297991 |
|  | 0.5 | 0.391320905 | 0.82424239 | 0.23188406 |
|  | 0.75 | 0.374094161 | 0.86730463 | 0.32443427 |
|  | 1 | 0.352954702 | 0.83125997 | 0.43096873 |
|  | 1.25 | 0.30595425 | 0.88421053 | 0.50470379 |
|  | 1.5 | 0.269302858 | 0.75342903 | 0.50275446 |
|  | 1.75 | 0.256993293 | 0.76406166 | 0.4781761 |
|  | 2 | 0.233224222 | 0.70696437 | 0.40961098 |
|  | 2.25 | 0.194711495 | 0.66241362 | 0.35952199 |
|  | 2.5 | 0.214697251 | 0.52099946 | 0.16221714 |
|  | 2.75 | 0.05818612 | 0.0523126 | 0.02873125 |
|  | 3 | 0.004434742 | 0.00988836 | 0.02313755 |
| P7 | -3 | 0.147155398 | 0.04074703 | 0.48445985 |
|  | -2.75 | 0.100371793 | 0.04046406 | 0.58437972 |
|  | -2.5 | 0.210900339 | 0.05800792 | 0.78787069 |
|  | -2.25 | 0.246710523 | 0.13582343 | 0.92332354 |
|  | -2 | 0.212307074 | 0.18392756 | 0.89633983 |
|  | -1.75 | 0.230793503 | 0.21844935 | 0.77771841 |
|  | -1.5 | 0.178072775 | 0.17713639 | 0.61759727 |
|  | -1.25 | 0.252926316 | 0.12903226 | 0.56995279 |
|  | -1 | 0.228922192 | 0.17289191 | 0.35158963 |
|  | -0.75 | 0.235044594 | 0.11375212 | 0.34010152 |
|  | -0.5 | 0.332388763 | 0.46557252 | 0.33823137 |
|  | -0.25 | 0.35051135 | 0.70335789 | 0.13652151 |
|  | 0 | 0.332383264 | 0.68081497 | 0.05583756 |
|  | 0.25 | 0.382901105 | 0.57366537 | 0.04328079 |
|  | 0.5 | 0.407320411 | 0.75183922 | 0.11675127 |
|  | 0.75 | 0.34260286 | 0.81777023 | 0.28185947 |
|  | 1 | 0.295034908 | 0.43623848 | 0.33609404 |
|  | 1.25 | 0.261702203 | 0.25693265 | 0.53406361 |
|  | 1.5 | 0.243400095 | 0.17968308 | 0.66176863 |
|  | 1.75 | 0.228665121 | 0.16327108 | 0.66328258 |
|  | 2 | 0.215174099 | 0.16893039 | 0.79267967 |
|  | 2.25 | 0.212625485 | 0.17062818 | 0.80550361 |
|  | 2.5 | 0.210344032 | 0.15393322 | 0.76756612 |
| P8 | -2.75 | 0.279494573 | 0.15180809 | 0.85361071 |
|  | -2.5 | 0.329596072 | 0.2273541 | 0.87937896 |
|  | -2.25 | 0.283731463 | 0.18331543 | 0.87961926 |
|  | -2 | 0.328810192 | 0.22162549 | 0.85758126 |
|  | -1.75 | 0.267410102 | 0.25241676 | 0.76356179 |
|  | -1.5 | 0.280636692 | 0.51843899 | 0.79235649 |
|  | -1.25 | 0.26875909 | 0.62382148 | 0.68259439 |
|  | -1 | 0.282720704 | 0.63802363 | 0.49606733 |
|  | -0.75 | 0.387669675 | 0.59529771 | 0.30228151 |
|  | -0.5 | 0.426784322 | 0.65497075 | 0.19364978 |
|  | -0.25 | 0.473717081 | 0.64076864 | 0.12366867 |
|  | 0 | 0.428185012 | 0.70235113 | 0.17586526 |
|  | 0.25 | 0.457400311 | 0.64960018 | 0.2607546 |
|  | 0.5 | 0.504926679 | 0.74257071 | 0.34538165 |
|  | 0.75 | 0.486622148 | 0.71273423 | 0.60696876 |
|  | 1 | 0.336541592 | 0.43967061 | 0.79018291 |
|  | 1.25 | 0.332544036 | 0.2244898 | 0.75387151 |
|  | 1.5 | 0.322722928 | 0.1811672 | 0.76624041 |
|  | 1.75 | 0.260733592 | 0.14070892 | 0.81766587 |
|  | 2 | 0.251730435 | 0.11027569 | 0.81288996 |
|  | 2.25 | 0.212623819 | 0.1160043 | 0.60804917 |
|  | 2.5 | 0.246221799 | 0.03222342 | 0.19709547 |
|  | 2.75 | 0.166075529 | 0.01682778 | 0.03754387 |
| P9 | -2.5 | 0.22906152 | 0.0947044 | 0.60327362 |
|  | -2.25 | 0.278658302 | 0.07817349 | 0.57463394 |
|  | -2 | 0.292057977 | 0.15522781 | 0.7544585 |
|  | -1.75 | 0.334809895 | 0.26603599 | 0.84615148 |
|  | -1.5 | 0.365609742 | 0.38031063 | 0.8198357 |
|  | -1.25 | 0.357135993 | 0.44158176 | 0.78148684 |
|  | -1 | 0.386305073 | 0.53111309 | 0.78182389 |
|  | -0.75 | 0.356475343 | 0.7336943 | 0.56368893 |
|  | -0.5 | 0.397277461 | 0.67108995 | 0.29576957 |
|  | -0.25 | 0.399277681 | 0.60560798 | 0.13258873 |
|  | 0 | 0.439550195 | 0.50780953 | 0.04059649 |
|  | 0.25 | 0.430504017 | 0.53570018 | 0.08681748 |
|  | 0.5 | 0.50547453 | 0.59179583 | 0.18522031 |
|  | 0.75 | 0.456847506 | 0.67333022 | 0.3520749 |
|  | 1 | 0.383016183 | 0.84322252 | 0.66580263 |
|  | 1.25 | 0.438803902 | 0.60784634 | 0.73687719 |
|  | 1.5 | 0.341207151 | 0.42158534 | 0.76675371 |
|  | 1.75 | 0.344983108 | 0.3584478 | 0.7427635 |
|  | 2 | 0.265697598 | 0.24540079 | 0.70257692 |
|  | 2.25 | 0.252761604 | 0.24177402 | 0.58779184 |
|  | 2.5 | 0.206252967 | 0.29339249 | 0.53860864 |
|  | 2.75 | 0.224402195 | 0.30448396 | 0.4659224 |
|  | 3 | 0.119712235 | 0.20503313 | 0.26652935 |
| P10 | -2.25 | 0.209854473 | 0.24443375 | 0.87035873 |
|  | -2 | 0.273668998 | 0.27144661 | 0.92516342 |
|  | -1.75 | 0.268455626 | 0.2994784 | 0.92358844 |
|  | -1.5 | 0.281125329 | 0.36155614 | 0.84610535 |
|  | -1.25 | 0.323315495 | 0.42349689 | 0.80591568 |
|  | -1 | 0.33252686 | 0.4860841 | 0.76068615 |
|  | -0.75 | 0.299305521 | 0.74951416 | 0.77561497 |
|  | -0.5 | 0.379917762 | 0.90061279 | 0.57412946 |
|  | -0.25 | 0.359477949 | 0.91199559 | 0.31777549 |
|  | 0 | 0.401216028 | 0.78743277 | 0.14192727 |
|  | 0.25 | 0.427434963 | 0.89772573 | 0.3014276 |
|  | 0.5 | 0.393935853 | 0.88121222 | 0.4114154 |
|  | 0.75 | 0.422842148 | 0.83789134 | 0.54968672 |
|  | 1 | 0.283111133 | 0.74068051 | 0.68890427 |
|  | 1.25 | 0.239169286 | 0.48730761 | 0.69164461 |
|  | 1.5 | 0.20379563 | 0.31853953 | 0.76941032 |
|  | 1.75 | 0.179644031 | 0.24667679 | 0.7665444 |
|  | 2 | 0.235474179 | 0.28408686 | 0.74238627 |
|  | 2.25 | 0.166936615 | 0.23250815 | 0.59671234 |
|  | 2.5 | 0.142482422 | 0.1511656 | 0.47598459 |
|  | 2.75 | 0.084179166 | 0.08215628 | 0.14910906 |
|  | 3 | 0.189957366 | 0.06717238 | 0.04856998 |
|  | 3.25 | 0.236698327 | 0.08307399 | 0.01738852 |
| P11 | -2.25 | 0.291860965 | 0.02501624 | 0.73205742 |
|  | -2 | 0.302637418 | 0.03638726 | 0.68941491 |
|  | -1.75 | 0.362740266 | 0.07050032 | 0.54134137 |
|  | -1.5 | 0.345647075 | 0.15074724 | 0.5281625 |
|  | -1.25 | 0.338155036 | 0.11825861 | 0.4630236 |
|  | -1 | 0.351543436 | 0.31069948 | 0.3601108 |
|  | -0.75 | 0.379338137 | 0.66742463 | 0.53756401 |
|  | -0.5 | 0.40871327 | 0.78080994 | 0.81339713 |
|  | -0.25 | 0.527818833 | 0.81059129 | 0.44489212 |
|  | 0 | 0.546395536 | 0.83181709 | 0.22185847 |
|  | 0.25 | 0.495692192 | 0.80290221 | 0.23369428 |
|  | 0.5 | 0.513575758 | 0.69471507 | 0.36397215 |
|  | 0.75 | 0.440070524 | 0.70218746 | 0.51498363 |
|  | 1 | 0.394867865 | 0.55869591 | 0.63476875 |
|  | 1.25 | 0.324513354 | 0.21258382 | 0.61269204 |
|  | 1.5 | 0.332205446 | 0.05295647 | 0.36993201 |
|  | 1.75 | 0.256017736 | 0.01234568 | 0.34701587 |
|  | 2 | 0.257978418 | 0.01267057 | 0.31478217 |
|  | 2.25 | 0.268810288 | 0.02794022 | 0.3659028 |
|  | 2.5 | 0.300693134 | 0.02988954 | 0.37043566 |
|  | 2.75 | 0.226469016 | 0.01689409 | 0.31931503 |
|  | 3 | 0.067868781 | 0.00617284 | 0.08939814 |
| P12 | -2.75 | 0.148055333 | 0.2149819 | 0.65241228 |
|  | -2.5 | 0.144851487 | 0.24227235 | 0.72331871 |
|  | -2.25 | 0.15540684 | 0.23001949 | 0.67690058 |
|  | -2 | 0.175645308 | 0.27151211 | 0.64912281 |
|  | -1.75 | 0.188831467 | 0.30632136 | 0.69371345 |
|  | -1.5 | 0.175535719 | 0.25006962 | 0.64144737 |
|  | -1.25 | 0.202287188 | 0.23141186 | 0.65387427 |
|  | -1 | 0.180909224 | 0.33221944 | 0.7221004 |
|  | -0.75 | 0.252407614 | 0.60772292 | 0.8702485 |
|  | -0.5 | 0.225592689 | 0.80933807 | 0.74622321 |
|  | -0.25 | 0.23857008 | 0.7207839 | 0.37573099 |
|  | 0 | 0.250146754 | 0.71725592 | 0.39656433 |
|  | 0.25 | 0.27795192 | 0.86800334 | 0.7677875 |
|  | 0.5 | 0.28113502 | 0.64847313 | 0.9273879 |
|  | 0.75 | 0.269095933 | 0.38280897 | 0.8373538 |
|  | 1 | 0.184367893 | 0.18323587 | 0.61306042 |
|  | 1.25 | 0.153561489 | 0.22472849 | 0.73586743 |
|  | 1.5 | 0.191194491 | 0.24227235 | 0.75816279 |
|  | 1.75 | 0.13114249 | 0.17460317 | 0.69407895 |
|  | 2 | 0.109067615 | 0.1539961 | 0.62646199 |
|  | 2.25 | 0.11009329 | 0.21303258 | 0.64839181 |
|  | 2.5 | 0.047172894 | 0.19242551 | 0.52266082 |
|  | 2.75 | 0.022781808 | 0.16402116 | 0.39729532 |
